# Gain-loss asymmetry in neural correlates of temporal discounting: An approach-avoidance motivation perspective

**DOI:** 10.1038/srep31902

**Published:** 2016-08-25

**Authors:** Yang-Yang Zhang, Lijuan Xu, Li-Lin Rao, Lei Zhou, Yuan Zhou, Tianzi Jiang, Shu Li, Zhu-Yuan Liang

**Affiliations:** 1 Key Laboratory of Behavioral Sciences & Magnetic Resonance Imaging Research Center, Institute of Psychology, Chinese Academy of Sciences, Beijing, 100101, China; 2 University of Chinese Academy of Sciences, Beijing, 100039, China; 3 National Laboratory of Pattern Recognition, Institute of Automation, Chinese Academy of Sciences, Beijing, 100190, China; 4 Brainnetome Center, Institute of Automation, Chinese Academy of Sciences, Beijing, 100190, China; 5 Key Laboratory for NeuroInformation of the Ministry of Education, School of Life Science and Technology, University of Electronic Science and Technology of China, Chengdu, 625014, China

## Abstract

Gain-loss asymmetry in temporal discounting (i.e., when individuals discount gains more than losses) has been implicated in numerous problematic and addictive behaviors, resulting in enormous personal and societal costs. On the basis of findings from a previous study, we speculated that approach-avoidance motivation would modulate gain-loss asymmetry. To test this speculation, we examined the effects of motivation on gain-loss asymmetry by analyzing functional connectivity. We found that approach and avoidance motivation were negatively associated with functional connectivity between the medial orbitofrontal cortex (MOFC) and the dorsolateral prefrontal cortex and functional connectivity between the MOFC and the posterior parietal cortex (PPC) in the gain domain. Only avoidance motivation was found to be positively associated with functional connectivity between the medial prefrontal cortex (MPFC) and the posterior cingulate cortex (PCC) as well as between the MPFC and the insula in the loss domain. Our findings suggest that the relationships of approach-avoidance motivation and neural correlates yielded an asymmetrical pattern between the gain and loss domains in temporal discounting. Thus, we provide new insight into understanding gain-loss asymmetry in temporal discounting.

When engaging in temporal discounting, humans typically discount future gains more than future losses, a phenomenon known as gain-loss asymmetry^1^. Excessive discounting in such intertemporal choices is implicated in several problematic and addictive behaviors, leading to enormous costs for both individuals and society^2^,^3^,^4^,^5^,^6^. Several explanations for gain-loss asymmetry have been advanced (e.g., a more elastic value function for loss^1^, a more anticipatory utility of loss^7^, anticipatory emotions^8^, etc.). Although these proposed explanations for gain-loss asymmetry differ in terms of mechanisms, they all suggest that gain-loss asymmetry may be modulated by motivation. Nonetheless, the modulation effects of motivation on gain-loss asymmetry and its neural correlates remain poorly understood. Studies have consistently shown that several functional brain networks (such as the valuation and cognitive control networks) play important roles in temporal discounting (for a review, see ref. 9). Thus, the present study examined the impacts of motivation on gain-loss asymmetry by analyzing functional connectivity.

Among all the possible explanations for gain-loss asymmetry, there are two key factors: valuation and emotion. From the valuation perspective, some researchers have suggested that gain-loss asymmetry might result from a value function pieced together from two independent segments (one for losses and one for gains) that connect at a reference point^1^,^7^ and that the value function for losses is steeper and more elastic than it is for gains^1^. Thus, the loss in value associated with a given monetary loss would exceed the gain in value produced by a monetary gain of the same absolute size, resulting in gain-loss asymmetry^1^. Previous research has reported that emotions – and particularly negative emotions – affected the valuation of loss outcome^10^,^11^,^12^,^13^. For example, depressive patients (who often hold negative emotions) had higher discounting rates than healthy adults for future rewards^11^,^13^ and immediate loss^13^. Thus, from an emotional perspective, Caplin and Leahy^8^ proposed the psychological expected utility model, which includes anticipatory emotions. In contrast with positive outcomes, negative outcomes increase anticipatory anxiety. As an extended waiting period before a negative outcome will generate a markedly greater buildup of anxiety than a shorter waiting period, it is thus better for individuals to experience losses immediately^8^. However, individuals extend periods during which they can savor the anticipation of desirable outcomes^8^.

Despite their differences, all the above explanations have one common factor, i.e., they all suggest that motivation might modulate gain-loss asymmetry. Based on a valuation perspective, motivation might influence decision-making through subjective valuation^14^. Approach motivation is positively correlated with a reward valuation system, such as the medial orbitofrontal gyrus^15^,^16^. Unlike individuals with low approach motivation, individuals with high approach motivation believe that different levels of gain are equally attractive^14^. By contrast, unlike individuals with low avoidance motivation, individuals with high avoidance motivation feel that different levels of loss are equally unattractive^14^. Alternatively, based on an emotional perspective, motivations are highly correlated with emotions^17^,^18^,^19^. Thus, approach motivation is responsible for the experience of positive feelings, such as hope, elation, and happiness^18^,^19^, whereas avoidance motivation leads to the experience of negative feelings, such as fear, anxiety, and frustration^20^. Carver and White found that approach motivation was positively correlated with self-reported levels of happiness when cues of impending rewards were presented^17^. By contrast, avoidance motivation was positively correlated with self-reported levels of nervousness when cues of impending punishment were presented. Individuals with high approach motivation were happier, and those with high avoidance motivation were more nervous^17^. In short, this evidence suggests that approach motivation may play a role in the gain domain, whereas avoidance motivation may play a role in the loss domain. Based on the foregoing, we speculated that approach-avoidance motivation might modulate gain-loss asymmetry.

Most research on temporal discounting using functional magnetic resonance imaging (fMRI) has shown that multiple brain regions are involved in temporal discounting^21^. Peters and Buechel indicated that valuation and cognitive control processes may act as essential components that are simultaneously involved in temporal discounting^9^. The main region facilitating the valuation process was found to be the ventromedial prefrontal cortex (VMPFC), whereas the main region facilitating the cognitive control process was found to be the dorsal lateral prefrontal cortex (DLPFC)^9^,^22^. Notably, Peters and Buechel based their theory on findings about the temporal discounting of gains^9^. Our previous study^23^ and the study by Tanaka *et al.*^24^ on the temporal discounting of losses showed that the brain regions specifically involved in the temporal discounting of losses included the posterior cingulate cortex (PCC), the posterior parietal cortex (PPC), the medial prefrontal cortex (MPFC)^23^, the insula and the thalamus^23^,^24^, suggesting that the loss domain should not be disregarded.

How the systems or brain regions discussed above interact with one another is essential to understanding the neural mechanism of temporal discounting^9^,^21^,^25^. Recently, a small number of studies have reported that the interactions between brain regions might predict individuals’ temporal discounting behavior in both healthy adults and other samples. For example, Hare *et al.*^26^ found that the interaction between the DLPFC and the VMPFC increased when participants selected the delayed options. These authors found that the resting-state functional connectivity between money networks (such as the VMPFC and the PCC) and time networks (such as the DLPFC) was negatively correlated with temporal discounting rates^27^. Moreover, the increased connectivity between the prefrontal cortex and the reward-processing regions was associated with steeper discounting in children with attention-deficit/hyperactivity (ADHD) disorder^28^, and greater integration of the left fronto-insular cortex with the left fronto-parietal network was associated with a steeper discounting rate among smokers^29^. Taken together, these findings hint to us that interactions between brain regions may also play a critical role in gain-loss asymmetry for temporal discounting.

Thus, the present study employed functional connectivity analysis to examine the role of approach-avoidance motivation in gain-loss asymmetry regarding temporal discounting. We hypothesized that approach motivation would negatively correlate with functional connectivity between seed regions (such as valuation and cognitive control regions) in the gain domain, given that approach motivation is positively correlated with the reward valuation system^14^,^15^,^16^ and that valuation was negatively correlated with cognitive control regions^22^. Moreover, we hypothesized that avoidance motivation would positively correlate with functional connectivity between seed regions (such as valuation and negative emotions regions) in the loss domain, given that the valuation of loss outcomes invoked negative emotions^10^,^11^,^12^,^13^ and that avoidance motivation positively correlated with negative emotions^20^. To test these hypotheses, we measured the levels of approach and avoidance trait motivation of the participants in our previous study^23^. By correlating the motivation score with the published fMRI data^23^, we examined whether approach and avoidance motivation during temporal discounting could 1) modulate gain-loss asymmetry and/or 2) modulate functional connectivities between brain regions identified in gain-loss asymmetry. Specifically, we selected significant clusters of activation in temporal discounting tasks used in our previously published dataset^23^ as seed regions, and then performed a seed-based functional connectivity analysis. In contradistinction to our previous study that focused on brain regions selectively responding to discounting future losses and not to discounting future gains, the present study focused on whether the relationship between approach-avoidance motivation and interactions among previously identified regions differed in the gain and loss domains.

## Results

### Behavioral results

Based on the behavioral choices results and following Ericson *et al.*^30^, we estimated a hyperbolic temporal discounting rate for each participant via maximum likelihood using a constrained optimization suite in R that implements the L-BFGS-B algorithm^31^ in the gain and loss domains. The hyperbolic function^32^ in our estimation is:


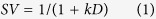


where SV is the subjective value of future outcomes, D is the delay of future outcomes (in months) and *k* is the temporal discounting rate. The mean temporal discounting rate in the gain domain was 0.28 ± 0.38 with a range of 0.01–1.35. The mean temporal discounting rate in the loss domain was 2.13 ± 2.20 with a range of 0.01–10.00.

Based on the mean scores of approach (*M* ± *SD*: 2.33 ± 1.46, range: 0–6) and avoidance motivation (*M* ± *SD*: 6.67 ± 3.68, range: 2–13), the participants were divided into two groups. To test whether the gain-loss asymmetry in the temporal discounting rates existed, we conducted two mixed ANOVAs with approach or avoidance motivation groups (high/low) respectively as between-subjects factors. The analysis showed that the temporal discounting rates were higher in the loss domain than in the gain domain, *F*_approach_(1, 16) = 9.31, *p* = 0.008, and *F*_avoidance_(1, 16) = 11.23, *p* = 0.004. Given that the heterogeneity of behavioral gain-loss asymmetry might raise from variety of participants’ motivations, we conducted Bonferroni *post hoc* tests and found a discrepant effect of approach or avoidance motivation on gain-loss asymmetry in temporal discounting rates. Specifically, for the approach motivation, in low motivation group, the temporal discounting rates in loss domain (*M* = 2.19) were significantly higher than that in gain domain (*M* = 0.22), *p* = 0.011, whereas there was no such effect in high approach motivation group, *p* = 0.11. For the avoidance motivation, the temporal discounting rates in loss domain were significantly or marginal significantly higher than that in gain domain in low (*M*_loss_ = 2.54, *M*_gain_ = 0.44, *p* = 0.016) or high motivation group (*M*_loss_ = 1.73, *M*_gain_ = 0.13, *p* = 0.057). These results indicated that the changing of the motivation level influenced the existence of gain-loss asymmetry of temporal discounting rates.

To further investigate the role of approach-avoidance motivation in gain-loss asymmetry, we also computed the Pearson correlations between approach motivation, avoidance motivation and temporal discounting rates. In the gain domain, the temporal discounting rate marginally significantly correlated with approach motivation (*r* = 0.45, *p* = 0.061) and did not correlate with avoidance motivation (*r* = −0.17, *p* = 0.492), whereas we did not find a significant correlation between the temporal discounting rate and motivations in the loss domain (approach motivation: *r* = 0, *p* = 0.999; avoidance motivation: *r* = −0.13, *p* = 0.597). These results indicated that in the gain domain the higher were individuals’ approach motivation, the delayed outcomes were discounted more steeply.

### Neuroimaging results

**Figure 1:**
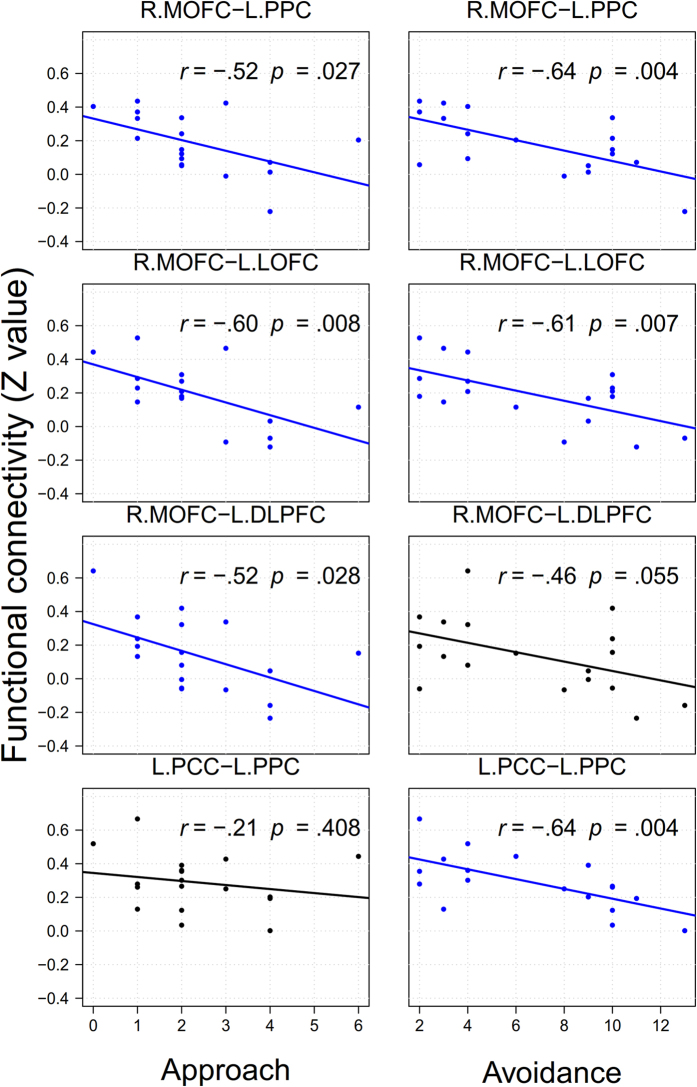
The correlation of functional connectivity with approach or avoidance motivation across participants in the G-TD condition.

**Figure 2:**
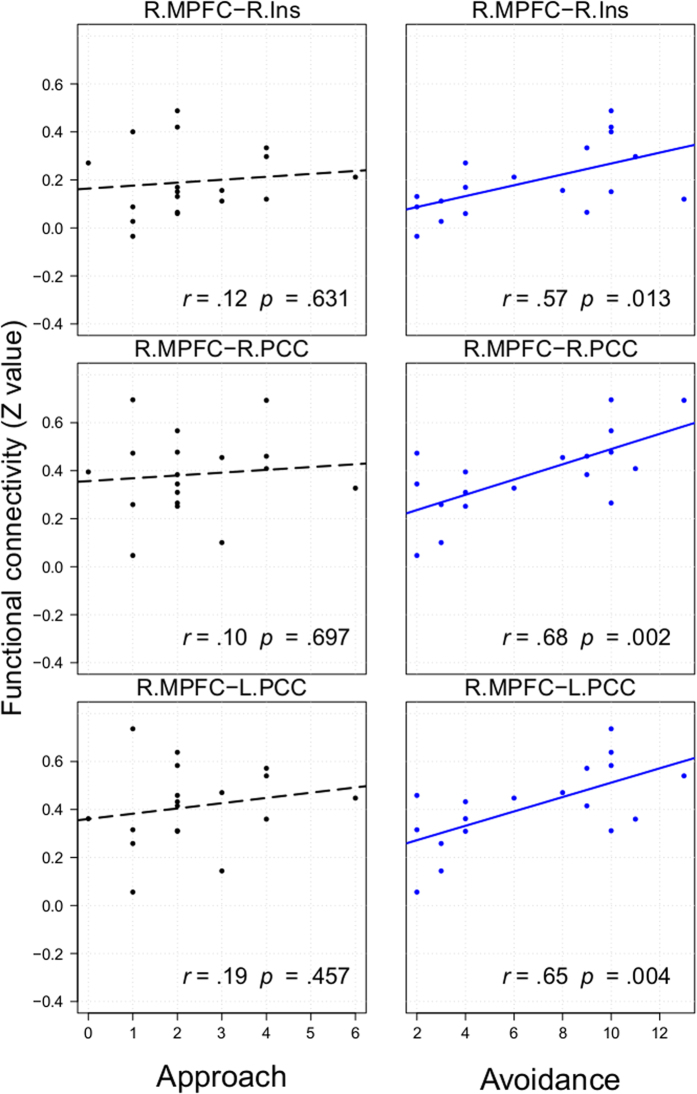
The correlation of functional connectivity with approach or avoidance motivation across participants in the L-TD condition.

The results of the functional connectivity analysis addressed whether task-induced patterns of connectivity in each task varied across individuals modulated by approach-avoidance motivation. During the G-TD, individual differences in approach motivation were significantly and negatively correlated with functional connectivity between the MOFC and the DLPFC, the MOFC and the LOFC and between the MOFC and the PPC (Fig. 1 and Table S1). We also found negative correlations whereby low levels of avoidance motivation were related to higher levels of functional connectivities between the MOFC and the LOFC, the MOFC and the PPC, and functional connectivity between the PCC and the PPC in the G-TD (Fig. 1 and Table S1). Participants with higher avoidance motivation levels presented higher levels of functional connectivity between the MPFC and the PCC and between the MPFC and the insula during the L-TD (Fig. 2 and Table S1). To test the difference between two correlation coefficients, we transformed Pearson’s correlations of motivation and functional connectivities into Fisher’s *z* test (one-tailed). Except for L.PCC-L.PPC, *z*(18) = 1.49, *p* = 0.07, the correlations in the gain domain between approach and avoidance motivation had no significant differences: R.MOFC-L.PCC, *z*(18) = 0.50, *p* = 0.31; R.MOFC-L.LOPFC, *z*(18) = 0.04, *p* = 0.48; and R.MOFC-L.DLPFC, *z*(18) = −0.22, *p* = 0.41. In the loss domain, the correlations between approach and avoidance motivation had significant or partially significant differences: R.MPFC-R.Ins, *z*(18) = 1.44, *p* = 0.07; R.MPFC-R.PCC, *z*(18) = 2, *p *= 0.02; and R.MPFC-L.PCC, *z*(18) = 1.6, *p* = 0.05. These results indicated that the effects of approach/avoidance motivation on the neural correlates in temporal discounting were different in the loss domain and were more likely to be similar in the gain domain.

## Discussion

The present study investigated the effects of approach-avoidance motivation on gain-loss asymmetry in neural correlates of temporal discounting. Our behavioral results indicated that the effect of motivations showed an idiosyncratic pattern on gain-loss asymmetry of temporal discounting rate: the gain-loss asymmetry effect appeared in low approach motivation group, and in both high and low avoidance motivation group. Meanwhile, only approach motivation marginally positively correlated with temporal discounting rates in the gain domain. Our neuroimage results showed that in the gain domain, approach motivation was negatively associated with functional connectivities between the MOFC and the DLPFC, between the MOFC and the LOFC and between the MOFC and PPC, avoidance motivation was negatively associated with functional connectivities between the MOFC and the LOFC, between the MOFC and the PPC, and between the PCC and the PPC. However, only avoidance motivation was positively associated with functional connectivity between the MPFC and the PCC and between the MPFC and the insula in the loss domain. These results suggest that the relationships between approach-avoidance motivation and neural correlates showed an asymmetrical pattern between the gain and the loss domains in temporal discounting.

The functional connectivity results were partially consistent with our hypothesis: we found that approach motivation was negatively correlated with functional connectivity between the MOFC, the PCC, the DLPFC, and the PPC in the gain domain. This result was also consistent with the behavioral results that approach motivation is marginally positively correlated with the temporal discounting rate in the gain domain. The MOFC and the PCC have been implicated in reward valuation processing^9^, whereas the DLPFC and the PPC have been implicated in cognitive control processing^9^,^23^. Peters and Buechel proposed that the DLPFC may influence value signals in valuation regions (i.e., the MPFC) through functional connectivity during temporal discounting^9^. Our results indicated that increasing approach motivation levels can reduce functional connectivity between cognitive control and valuation processing. In addition – and also consistent with our predictions – we found that avoidance motivation was positively correlated with functional connectivities between the MPFC, the PCC, and the insula in the loss domain. Previous studies have shown that anticipated or experienced losses lead to activation in the insula^33^ and that the insula has been consistently associated with negative emotions^23^,^34^,^35^,^36^. This result indicated that increasing avoidance motivation levels can enhance functional connectivity between emotion and valuation processing in the loss domain. However, because we failed to find a correlation between avoidance motivation and the temporal discounting rate in the loss domain, this explanation is speculative and further studies are required to validate it.

Nonetheless, we found avoidance motivation to be negatively correlated with functional connectivity between the MOFC, the PPC, and the PCC in the gain domain, which we did not hypothesize. This result suggests that avoidance motivation may influence functional connectivity during valuation processing in the gain domain perhaps because individuals with higher levels of avoidance motivation are less sensitive to rewards^14^ and are thus more likely to resist the temptation to obtain money as soon as possible. This interpretation is supported by previous study findings that showed that reward-related activation levels were lower in individuals with higher levels of avoidance motivation^16^, but this initial interpretation requires further investigation. Notably, we did not find such correlations in the behavioral results between avoidance motivation and the temporal discounting rate in the gain domain, but this result might be due to the relatively small sample size of our study. The fMRI data may yield a more sensitive probe of the motivation effect than a behavioral evaluation using the same sample size.

Our results showed how the asymmetrical effect of approach/avoidance motivation affected the neural correlates of temporal discounting in both the gain and the loss domains. Specifically, both approach and avoidance motivation contributed similarly to the neural correlates of temporal discounting in the gain domain. By contrast, only the avoidance motivation contributed to the neural correlates of temporal discounting in the loss domain. These results highlighted the substantial role of avoidance motivation in the temporal discounting of loss. Given that individuals were much more sensitive to loss than to the same amount of gain^33^,^37^, avoidance motivation would affect the intertemporal choice process even when individuals confronted a relative small loss of money (less than ¥ 150) in the L-TD. However, when individuals faced the same amount of gain in the G-TD, such small gain might not have been sufficient to invoke a psychological response that would be commensurate with the same amount of loss, and the effects of approach/avoidance motivation would consequently be too weak to be distinguished from one another.

Our results also showed, on the behavioral level, how the idiosyncratic effect of approach/avoidance motivation affected the existence of gain-loss asymmetry of temporal discounting rates. Specifically, the gain-loss asymmetry of temporal discounting rates exist at different levels of the avoidance motivation. By contrast, the levels of approach motivation influenced the existence of gain-loss asymmetry of temporal discounting rates. In other words, this asymmetry effect appeared only in the participants with low motivation level. Notably, due to the relatively small sample size of our study, further studies are required to validate these effects.

Our findings contribute to the theoretical literature in terms of understanding the sign effect in temporal discounting by highlighting the role of approach-avoidance motivation in temporal discounting. Compared with previous fMRI studies that have investigated the mechanism of the sign effect from the perspective of emotion^23^ or loss aversion^24^, the present study uncovered motivational asymmetry underlying the sign effect from the perspective of motivation. Xu *et al.*^23^ argued that the sign effect was caused by negative emotions evoked by loss events as reflected by the insular activity. Tanaka *et al.*^24^ argued that the sign effect was caused by loss aversion and the ‘dread effect’, which might be reflected by insular and striatal activity, respectively. From a new perspective of motivation, our findings suggest that avoidance motivation increased the neural interaction between previously reported negative emotion regions and a crucial system in temporal discounting, the valuation network. This interpretation of motivation asymmetry may extend our understanding of the neural mechanisms underlying the sign effect.

Our findings also have potential practical application. Based on the mechanism of approach-avoidance motivation asymmetry, our results suggest that interventions of intertemporal choice must account for the various roles of approach/avoidance motivation in different domains. For example, to meet sustainable development objectives from a gain perspective, policy-makers might make efforts to modulate approach and avoidance motivation through attention training or emotional regulation to corresponding positive or negative outcomes^38^,^39^, in turn influencing individuals’ preferences in intertemporal choices. By contrast, to prevent environmental degradation from a loss perspective, environmental protection departments may merely take note of the effects of avoidance motivation on intertemporal choices. We must also note that a relatively small sample size was an inherent limitation of this study and that we have re-analyzed a previously collected dataset^23^. More samples are needed in future studies to enhance our conclusions.

In summary, our results suggest that both approach and avoidance motivation can modulate valuation processing of temporal discounting in the gain domain, whereas avoidance motivation can modulate valuation processing and emotional processing of temporal discounting in the loss domain. The present study thus provides new insights into the gain-loss asymmetry associated with temporal discounting.

## Method

### Participants

Twenty healthy, right-handed Chinese graduate students (10 females; mean age of 25.0 ± 1.7 years) were recruited. Two participants presenting excessive head motion were excluded from further analysis. All the participants had normal or corrected-to-normal vision, no history of neurological or psychiatric disorders, and signed written informed consent forms. This study was approved by the Institutional Review Board of Beijing MRI Center for Brain Research, and its methods were implemented in accordance with the approved guidelines.

### Experimental Task

**Figure 3:**
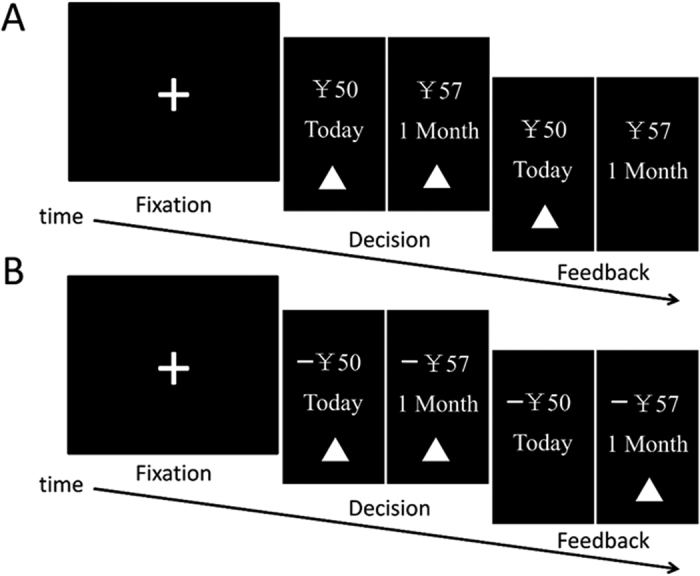
Illustration of a trial used in the experiment. (**A**) Trial structure for a temporal discounting task involving gain (G-TD). (**B**) Trial structure for a temporal discounting task involving loss (L-TD).

A temporal discounting task similar to that presented by Green and Myerson^40^ was used. The procedure involved two tasks: a temporal discounting task involving gains (G-TD) and a temporal discounting task involving losses (L-TD)^23^. Each task involved 42 trials. During each trial, participants were instructed to make a selection between two monetary options: a smaller amount that would be paid out sooner (rang: ¥ 13– ¥ 110; delay: 0, 0.5 and 1 month, e.g., “ ¥ 50 today”) and a larger amount that would be paid out later (rang: ¥ 20– ¥ 149; delay: 0.5, 1 and 2 months, e.g., “ ¥ 75 in 1 month”). For the L-TD, a “-” sign was placed before monetary amounts, denoting that money would be lost at the corresponding time. Participants indicated their choices by pressing one of the two buttons corresponding to the locations of the options on the screen. After a participant gave a response, the corresponding result was presented for 2 s followed by a black screen that was shown for 10 s until a 2 s fixation period had passed, which signaled the start of the next trial. Before beginning the L-TD, participants were given an endowment of ¥ 150 to cover their potential losses. After each experiment, one trial was randomly selected from each task, and participants were reimbursed (gains and losses) at the time specified in the selected options. More information on the temporal discounting task used in this study is detailed in Fig. 3 and can also be found in our previous study^23^.

### Motivation assessments

Given that trait motivation reflects stable and dispositional individual differences in both approach and avoidance motivations^41^,^42^,^43^, we assessed participants’ approach and avoidance trait motivation levels using two subscales of Zuckerman-Kuhlman Personality Questionnaire (ZKPQ), i.e., the Impulsive subscale (Imp, 8 items, part of the Impulsive Sensation-seeking subscale of ZKPQ) and the Neuroticism-Anxiety subscale (N-Anx, 19 items)^44^. Previous research has reported that individuals with higher impulsive traits also discounted rewards more steeply in the gain domain^44^, and the effects were so stable and pervasive that delay discounting may be considered a personality trait^45^,^46^. Given this relationship, we used the Imp subscale to measure the impulsive trait, which is a widely used indicator of the approach personality trait^41^,^42^,^43^. This subscale measures a lack of planning and a tendency to act impulsively without thinking^44^. The N-Anx subscale is widely used to indicate the avoidance personality trait^42^ and measures emotional upset, tension, worry, fearfulness, obsessive indecision, lack of self-confidence, and sensitivity to criticism^44^,^47^. Throughout this paper, approach and avoidance motivation refer to the Imp and N-Anx scores, respectively. After it was translated into Chinese^48^, the ZKPQ was individually administered during a separate session after the scanning procedure was completed.

### fMRI data acquisition and Preprocessing

Imaging data were collected using a 3.0 Tesla Siemens MRI scanner. Whole-brain functional scans were collected in 26 axial slices using an echo-planar imaging (EPI) sequence (TR/TE = 2000/30 ms, flip angle (FA) = 90^o^, field of view (FOV) = 19.2 cm, matrix = 64 × 64, thickness = 3 mm, gap = 1 mm). Two functional runs were collected for each participant using a 10-min T1-weighted anatomical scan intervening between the two runs.

Data were preprocessed and analyzed using SPM2 software (Wellcome Department of Imaging Neuroscience, London, UK). The first five images were discarded from the analyses. Functional images were corrected for differences in slice acquisition timing and then motion corrected. The images were then normalized to a standard EPI template for interparticipant comparison and spatially smoothed using an 8 mm full-width-at-half-maximum Gaussian kernel. The functional images were then detrended and high-pass filtered with a 128 s cutoff period for linear drift and low frequency fluctuation removal.

### Functional connectivity

The seed regions used in the functional connectivity analysis were selected based on significant clusters of activation that are identified through task-related regions analyses conducted in our previous study^23^. Regions-of-interest (ROIs) for the G-TD and L-TD were defined as the 6-mm-radius spheres centered in the peak activation foci of each task. We defined 13 ROIs as the main clusters of activation in the activation map for the G-TD group analysis, including the DLPFC, the LOFC, the PPC, the MOFC, the MPFC, the PCC, and the left ventral striatum. Seventeen ROIs, including the bilateral DLPFC, the LOFC, the PPC, the MPFC, the PCC, the ACC, the thalamus, the right striatum, the insula and the left SFG were defined as the main clusters of activation in the activation map for the L-TD. Illustrations of the location of the ROIs can be found in Fig. S1. Coordinates of the seed region foci are detailed in Tables 1 and 2.

Functional connectivity was measured by extracting the time series from all voxels in each seed region and then computing the correlation coefficients between the average time series in each pair of regions. The activation time course for each ROI was separately extracted for each participant, and head motion effects were removed through multiple regressions. To examine task-induced patterns of connectivity in each task, we extracted the entire time course of activity in each ROI for each task and multiplied that time course by a condition vector that was assigned a value of one for 6 TRs following the decision cue and a value of zero otherwise. The time course was then spliced and concatenated to include all the images acquired during the decision epoch, including additional images after the end of each epoch (to take advantage of the gradual decline in signal resulting from the delayed hemodynamic response). The time course included for each task was roughly equated across all participants.

We determined functional connectivity among the ROIs in the G-TD and L-TD to evaluate their interactions with one another. Pearson’s correlation coefficients were calculated for each pair of the averaged reference time series. Fisher’s r-to-z transformation was applied to the resulting set of correlations, which improved the normality of these correlation coefficients^49^. Individual z values for these correlation coefficients were then submitted to a random effect one-sample two-tailed *t*-test against a null hypothesis of no correlation to identify brain regions showing significant correlations within each group. To account for multiple comparisons, the Benjamini and Hochberg False Discovery Rate was applied^50^. To examine modulations of individual difference reflected in the interactions between these task-related regions, the relationship between the z values of the significant correlations and participants’ approach and avoidance motivation scores was accessed through a separate simple regression analysis.

## Additional Information

**How to cite this article**: Zhang, Y.-Y. *et al.* Gain-loss asymmetry in neural correlates of temporal discounting: An approach-avoidance motivation perspective. *Sci. Rep.* **6**, 31902; doi: 10.1038/srep31902 (2016).

## Supplementary Material

Supplementary Information

## Figures and Tables

**Table 1:** Coordinates of seed regions in G-TD.

Brain Regions	Abbreviations	BA	Cluster Size	Coordinates			T value
			(voxels)	x	y	z	
B Posterior Cingulate Cortex/Precuneus	R.PCC	31/7	30	3	−42	36	7.51
	L.PCC		32	−9	−60	33	5.79
B Medial Frontal Gyrus	R.MPFC	10	20	6	57	9	4.53
	L.MPFC		15	−3	57	−6	3.96
B Medial Orbital Gyrus	R.MOFC	11	20	3	48	−15	4.08
	L.MOFC		11	−3	45	−9	4.38
L Ventral Striatum*	L.VStr		5	−6	9	−6	4.16
R Inferior/Middle/Superior Frontal Gyrus	R.DLPFC	10/11/47	21	45	57	−9	10.26
	R.LOFC		18	27	69	−6	10.19
L Inferior/Middle/Superior Frontal Gyrus	L.DLPFC	10/11/47	27	−42	57	−9	11.45
	L.LOFC		31	−45	48	−15	8.69
R Posterior Parietal Cortex	R.PPC	40	31	54	−42	48	8.5
L Posterior Parietal Cortex	L.PPC	40	33	−51	−57	45	8

Coordinates of the peak voxel are reported in the Montreal Neurological Institute (MNI) space (*p* < 0.001, uncorrected; *p < 0.05, FDR corrected). B, bilateral; R, right; L, left; BA, Brodmann’s area; (x, y, z), coordinates of primary peak locations in the MNI space.

**Table 2:** Coordinates of seed regions in L-TD.

Brain Regions	Abbreviations	BA	Cluster Size	Coordinates			T value
			(voxels)	x	y	z	
B Posterior Cingulate Cortex/Precuneus	R.PCC	31/7	26	12	−51	36	6.97
	L.PCC		31	−3	−54	42	6.82
B Medial Frontal Gyrus/	R.MPFC	10/11/24/32	23	9	42	−6	5.6
Anterior Cingulate Cortex	L.MPFC		24	−3	51	−12	4.78
	R.ACC		30	3	36	15	6.28
	L.ACC		22	−6	30	15	5.61
L Superior Frontal Gyrus	L.SFG	9/10	23	−27	51	30	5.05
R Insula	R.Ins	13	15	42	−15	9	5.78
R Middle/Inferior/Superior Frontal Gyrus	R.LOFC	8/9/10	27	30	66	−6	9.74
	R.DLPFC	11/46/47	32	39	33	27	9.37
L Middle/Inferior/Superior Frontal Gyrus	L.LOFC	10/11	28	−45	54	−3	8.85
	L.DLPFC	46/47	33	−51	30	27	7.14
R Posterior Parietal Cortex	R.PPC	40/7	33	54	−45	48	11.18
L Posterior Parietal Cortex	L.PPC	40/7	31	−51	−54	30	8.39
R Thalamus/Striatum	R.Tha		32	24	−27	12	9.91
	R.Str		32	21	−3	6	8.61
L Thalamus	L.Tha		195	−21	−33	9	8.27

Coordinates of the peak voxel are reported in the Montreal Neurological Institute (MNI) space (*p* < 0.001, uncorrected). B, bilateral; R, right; L, left; BA, Brodmann’s area; (x, y, z), coordinates of primary peak locations in the MNI space.
