# Supplementary material for: Gain-loss asymmetry in neural correlates of temporal discounting: An approach-avoidance motivation perspective
Source: Sci Rep. 2016 Aug 25;6:31902. doi: 10.1038/srep31902 (PMC4997255; doi:10.1038/srep31902)
Supplement: Supplementary Information [file srep31902-s1.pdf]

Supplementary Information for

The gain-loss asymmetry in neural correlates of temporal discounting: An approach-avoidance motivation perspective

Yang-Yang Zhang[1,2,+], Lijuan Xu[3,+], Li-Lin Rao[1], Lei Zhou[1,2], Yuan Zhou[1], Tianzi Jiang[3,4,5], Shu Li[1], Zhu-Yuan Liang[1,*]

[1]Key Laboratory of Behavioral Sciences & Magnetic Resonance Imaging Research Center, Institute of Psychology, Chinese Academy of Sciences, Beijing 100101, China

[2]University of Chinese Academy of Sciences, Beijing 100039, China

[3]National Laboratory of Pattern Recognition, Institute of Automation, Chinese Academy of Sciences, Beijing 100190, China

[4]Brainnetome Center, Institute of Automation, Chinese Academy of Sciences, Beijing 100190, China

[5]Key Laboratory for NeuroInformation of the Ministry of Education, School of Life Science and Technology, University of Electronic Science and Technology of China, Chengdu 625014, China

[*] liangzy@psych.ac.cn

[+] These authors contributed equally to this work

Supplemental Tables

Supplemental Table S1. The significant correlations of the approach-avoidance scores and the strength of the functional connectivities in both G-TD and L-TD.

Region A	Region B	approach	avoidance
		CC	CC
**Gain**			
R.MOFC	L.DLPFC	-0.52*	
	L.LOFC	-0.60**	-0.61**
	L.PPC	-0.52*	-0.64***
L.PCC	L.PPC		-0.64***
**Lose**			
R.MPFC	R.Ins		0.57*
	R.PCC		0.68***
	L.PCC		0.65***

The abbreviations of brain regions were defined in Table S3 and S4. The numbers in the table is the Pearson's correlation coefficients, $^{*}p < 0.05$, $^{**}p < 0.01$; $^{***}p < 0.005$. Abbreviations: L, left; R, right; CC, correlation coefficient.

Supplemental Figures

Supplemental Figure S1

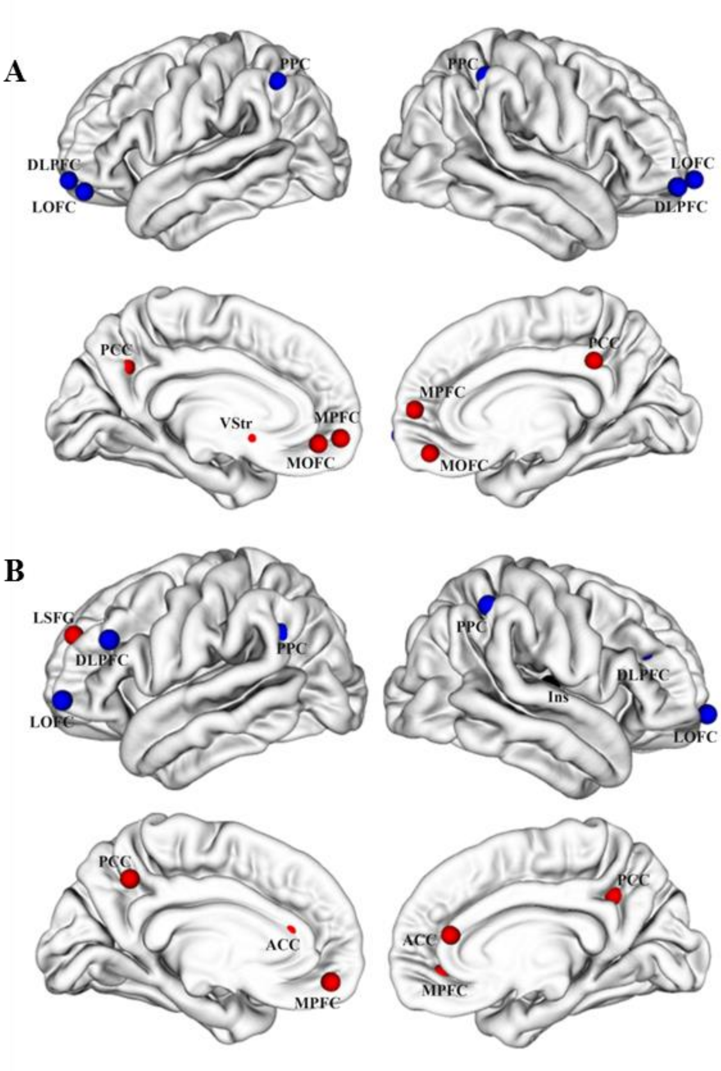

Fig. S1: Regions selected as the ROIs in the functional connectivity analyses. Red areas were regions preferentially activated by choices involving an immediate option. Blue areas were regions significantly activated across all decision epochs. (A) Seed regions in G-TD. (B) Seed regions in L-TD.

Supplemental Figure S2

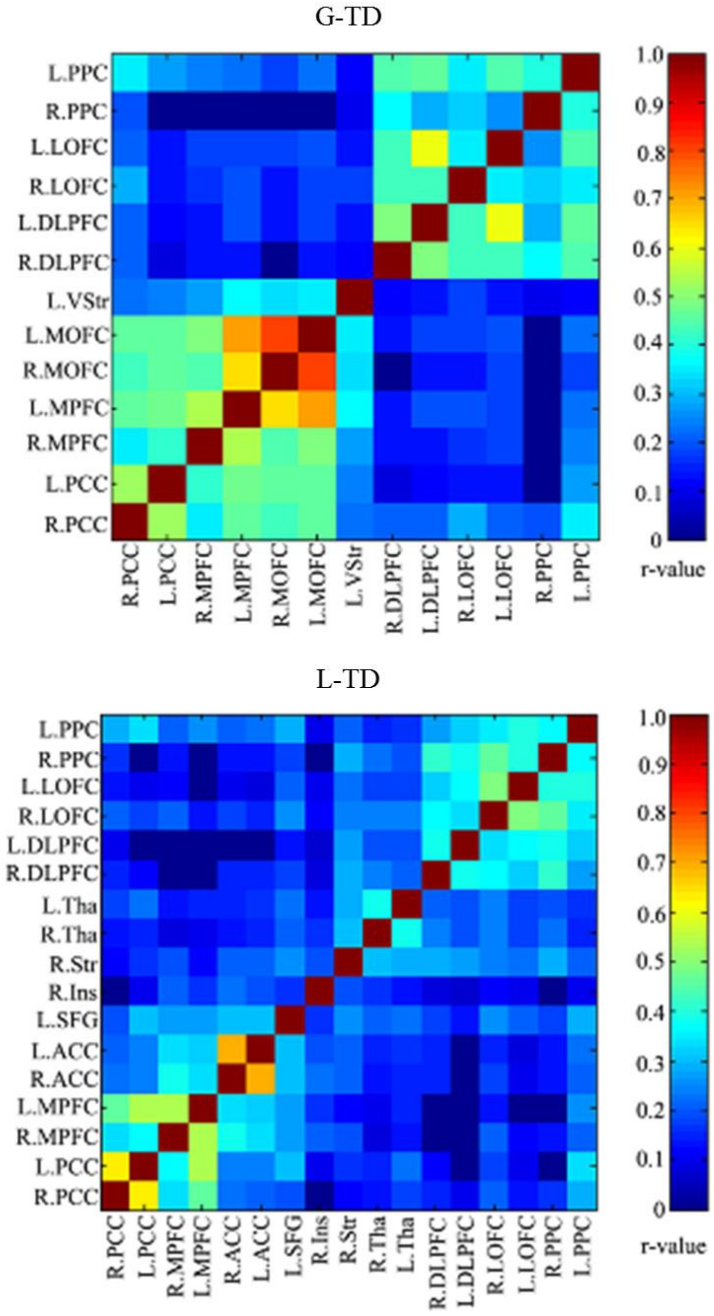

Fig. S2: Mean correlation matrices representing task-induced patterns of functional connectivity in G-TD and L-TD. Cooler boxes represent low-correlation coefficients between regions, whereas hotter boxes represent stronger correlations. Qualitatively, the regions of interest (ROIs) which were more activated by choices involving an immediate outcome showed dissociable correlations with ROIs which activated across all decision epochs.
